# The Interfaces Between Signal Transduction Pathways: IGF-1/mTor, p53 and the Parkinson Disease Pathway

**DOI:** 10.18632/oncotarget.759

**Published:** 2012-11-28

**Authors:** Arnold J. Levine, Chris R. Harris, Anna M. Puzio-Kuter

**Affiliations:** ^1^ Cancer Institute of New Jersey, UMDNJ, New Brunswick, N.J

**Keywords:** p53, parkinson disease, IGF-1, mTOR, RHEB

## Cell growth and division

The process of cell growth and division coordinates several input signals concerning the availability of nutrients in the environment of the cell. It then translates this information into intracellular activities that coordinate metabolic pathways to ensure sufficient energy sources, proper substrate concentrations and increased cell mass resulting in the production of two daughter cells. In the case of a single cell organism grown in culture, like yeast, the external glucose and amino acid concentrations are monitored. For multi-cellular organisms both glucose and amino acid concentrations are measured but in addition growth factors and cellular maintenance functions are monitored. This is accomplished by growth factor receptors that in turn initiate signal transduction pathways that regulate metabolic activity (IGF -1/mTor), and cell division (cell cycle regulators)[1-2]. These processes of cell growth and division require a high fidelity. A wide variety of stresses during cell division will lead to increases in error rates during DNA replication, DNA repair or chromosome segregation. One of the major responders (check points) to these types of stress (DNA damage, hypoxia, starvation for nutrients, etc) is the p53 pathway [2]. DNA damage is recognized by protein kinases such as ATM or ATR, which signal via phosphorylation to p53 and MDM-2, the ubiquitin ligase that promotes the degradation of p53 [3-4]. These rapid post-translational modifications inactivate MDM-2 and activate p53, which then promotes the transcription of selected genes. In this way p53 levels rise after cellular stresses. Stresses during the G-1 phase of the cell cycle are responded to by the p53 dependent transcription of the p21 gene [5]. The p21 protein binds to cyclin E-CDK-2 and inhibits it from stopping cell cycle progression in late G-1. Cells in the G-2/M phase of the cell cycle are blocked by p53 mediated transcription of 14-3-3 sigma which binds CDC-25c, keeping it in the cytoplasm and preventing this phosphatase from functioning in the cell nucleus [4]. If these types of cellular damage are not repaired the activated p53 protein can initiate cellular death programs (often depending upon the cell type or whether the cells are cancerous or not) resulting in apoptosis or cellular senescence. The p53 inducible genes, Bax, Puma and Noxa act at the mitochondia to help release cytochrome c, which in turn interacts with the p53 regulated gene product APAF-1 to start a caspase cascade leading to apoptosis [6-8]. In this way p53 acts as a cell division check point, eliminating mistakes that can lead to abnormal cell division and cancers. But p53 has more subtle functions when it is activated.

## The antagonistic relationship between the p53 and the IGF-1/mTor pathways

**Figure 1:**
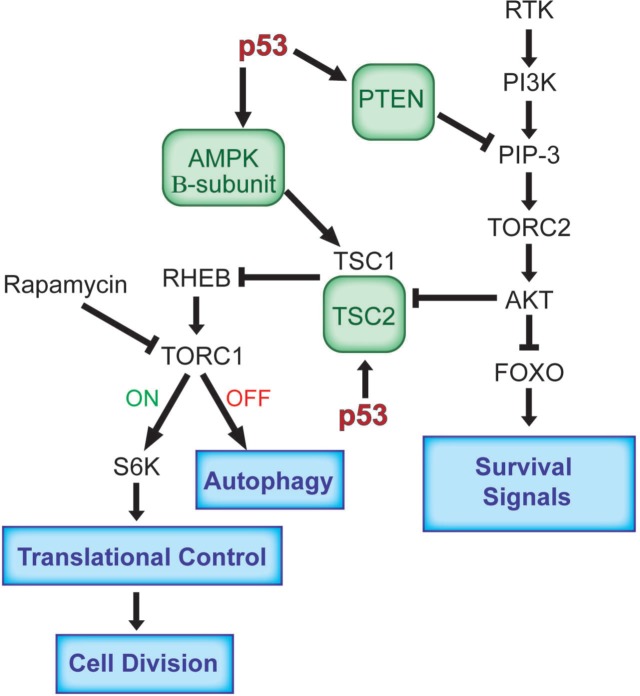
The antagonistic relationship between the p53 and the IGF-1/mTor pathways The activation of p53 in response to a lack of nutrient signals results in the enhanced transcription of Pten, TSC2 and AMPK (beta subunit), to block the functioning of the IGF-1/mTor pathways in transformed cells. This leads to a decrease in cell growth, which also mediates an inhibition of proliferation through p53 activation. Under conditions of glucose starvation, inactive TORC-1 (OFF) starts the process of autophagy, while under ample glucose conditions active TORC-1 (ON) phosphorylates its substrates leading to the regulation of translation promoting cell growth and division. Abbreviations: AMPK, AMP activated protein kinase; FOXO, Forkhead Box O; mTOR, mammalian target of rapamycin; mTORC, mammalian target of rapamycin complex; PI3K, phosphatidylinositol-3 kinase; PIP3, phosphatidylinositol 3,4,5-trisphosphate; PTEN, phosphatase and tensin homologue; Rheb, Ras homolog enriched in brain; S6K, ribosomal protein S6 kinase; TSC1, TSC2, tuberosclerosis complex.

The IGF-1 pathway is activated by the engagement of a wide variety of cellular tyrosine kinase growth receptors with their ligands. After dimerization of these receptors, phosphorylation and the binding of an adaptor protein, this complex attracts a PI3-kinase activity to the cellular membrane producing PIP-3 (phospho-inositol-3-phosphate), a ligand that activates the TORC2 complex, which in turn phosphorylates and activates AKT-1. This kinase moves into the cell nucleus and phosphorylates the FOXO transcription factors which then exit from the nucleus turning on or off the transcription of a number of gene products that enhance cell growth and division [9-11] (figure 1). At the same time several cellular sensors are monitoring the external and internal concentrations of glucose and several amino acids. This information is delivered to the AMP kinase. Under conditions of glucose starvation lower levels of ATP are produced and the cellular AMP concentration rises. The alpha subunit of the AMP kinase binds this AMP, the beta subunit connects this complex to the gamma subunit which is then an activated protein kinase. One of the substrates of AMP kinase is the TSC-1 and TSC-2 protein complex and the phosphorylation of these proteins enhances a GTPase activity that converts GTP (active form) to GDP (inactive form) that is associated with the RHEB G-protein [12]. An active RHEB is required for an active TORC1 protein kinase (Figure 1). An inactive TORC1 (resulting from glucose starvation) starts the process of autophagy where cellular components are sequestered into cytoplasmic vesicles and degraded in the lysozome so as to supply substrates for maintenance of the cell during starvation conditions [1]. In the presence of ample glucose, TORC1 is active and phosphorylates two substrates, S-6 kinase and 4EBP that regulate cellular translation favoring cell growth and division [13-15] The AKT-1 kinase connects these two pathways by phosphorylating TSC-1 and TSC-2 and inactivating them (the GTPase), promoting cell growth [12]. Thus these two interacting pathways cooperate and insure the proper levels of substrates and growth signals leading to the metabolic contributions to cell division (figure 1).

The utilization of glucose by the metabolic pathways of the cell can itself be regulated [2]. Under normal rates of cell division (24 hour division times) in an aerobic environment, which maintains a steady state of new cells formed and old cells dying, glucose is taken up into the cell and passes through glycolysis and is efficiently converted from pyruvate to acetyl-CoA to be burned in oxidative phosphorylation to CO_2_ and H_2_O in the mitochondria. This produces the maximal levels of ATP per mole of glucose with the cleavage of all of the carbon bonds in glucose. Under conditions of very rapid cell division with a net increase in cell number, such as occurs in early embryogenesis (6 hour division times), during wound healing or when T or B cell clones are expanded during an immune response, glucose is metabolically processed differently. Glucose is rapidly run through glycolysis building up pyruvate, which in turn is converted to lactate and excreted from the cell lowering the levels of oxidative phosphorylation. This process generates much lower levels of ATP per mole of glucose and to make up for this lower energy efficiency about ten times the level of glucose is transported into the cell, through regulated glucose (GLUT) transporters, and sent through glycolysis and other pathways like the pentose phosphate shunt. While this now provides sufficient energy sources it also moves the carbons from glucose into high concentrations of substrates for fatty acids, amino acids and nucleic acids. Thus a rapid proliferation leading to increased cell numbers switches metabolic utilization of glucose to a pathway first described by Warburg for cancer cells and termed the Warburg effect [16-17]. Several cellular functions can contribute to this switch in metabolic processing of glucose (using either high aerobic glycolysis or oxidative phosphorylation) and these include both proto-oncogenes (myc, AKT) and HIF-1 alpha favoring aerobic glycolysis. Several interacting tumor suppressor genes (PTEN, p53, TSC-1, TSC-2) favor oxidative phosphorylation [18-21]. This is why mutations in these genes often produce cancer cells with this altered metabolic pathway known as the Warburg effect (2).

When an activated p53 protein senses a stress that would interfere with normal progression through the cell cycle, it not only acts to stop cell cycle progression but it also shuts down the IGF-1 and mTor pathways [1,22-23] P53 mediated transcriptional activation of the PTEN gene produces the lipid phosphatase, PTEN, which degrades PIP-3 and the absence of PIP-3 inactivates AKT-1, TORC1 and 2 shutting down both arms of this signal transduction pathway [22,24]. P53 also transcribes the TSC-2 gene enhancing the GTPase that inhibits TORC1 and it transcribes the gene encoding the AMP kinase beta subunit increasing its concentration [23]. The net effect of an enhanced transcription of these three genes is that an active p53 blocks the functioning of the IGF-1/mTor pathways shutting down metabolic support for cell growth and division (figure 1). An activated p53 also promotes the use of oxidative phosphorylation and the complete burning of glucose, thus limiting substrates for cell division and antagonizing the Warburg effect. It does this by repressing transcription of Pdk2, the negative regulator of the pyruvate dehydrogenase complex, and thereby causes pyruvate to be converted into the TCA cycle substrate acetyl-CoA [25]. P53 also transcribes the SCO2 gene, which provides cytochrome c oxidase in complex 1 of the mitochrondria favoring oxidative phosphorylation [26]. P53 regulates the GLS-2 gene, which produces glutaminase-2 (GLS-2) that converts glutamine to glutamate and in the mitochondia provides more alpha-keto glutarate for the TCA cycle [27]. Finally p53 regulates the transcription of the Parkin gene, which also plays a role in slowing the Warburg effect [28].

## The role of reactive oxygen as a stressor in cell growth, division and death

**Figure 2:**
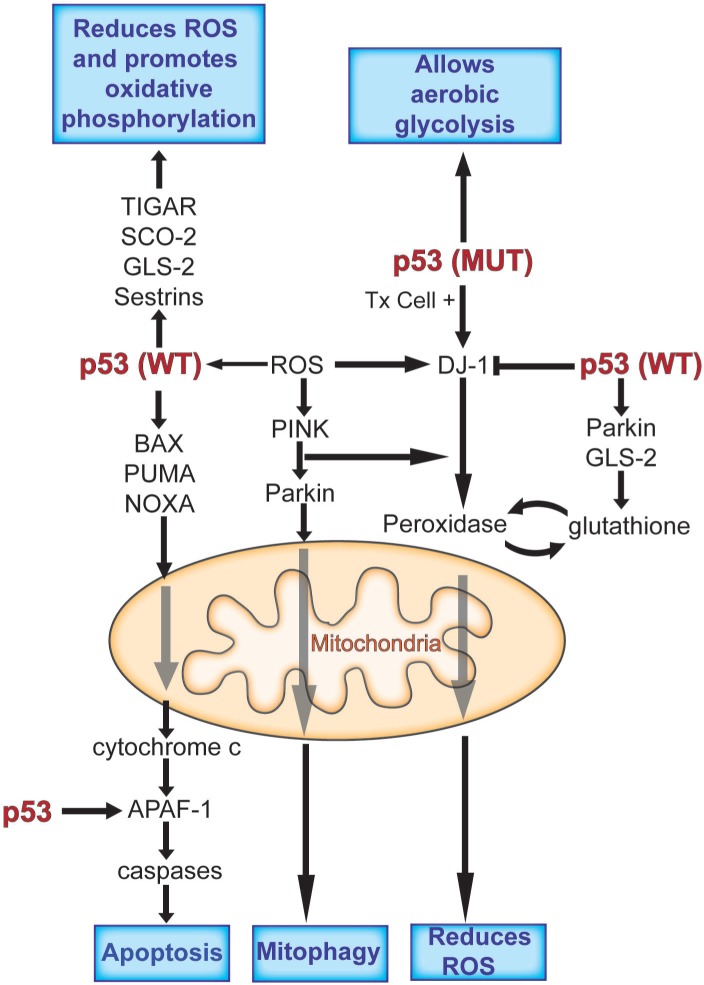
p53 functions in a complex network to mediate a cell's adaptation to stress p53 is able to reduce the flux through the glycolytic pathway and increase oxidative phosphorylation, and in doing so opposes the Warburg effect. p53 is also able to regulate oxidative stress, through increasing it or decreasing it. It can play an antioxidant role and protect cells from high levels of ROS, promoting cell survival or a pro-oxidant activity that contributes to removal of a damaged or stressed cell. Abbreviations: APAF-1, apoptotic peptidase activating factor 1; BAX, BCL2-associated X protein; GLS-2, glutaminase 2; PINK, pten-induced putative kinase 1; PUMA, *p53 upregulated modulator of apoptosis;* ROS, reactive oxygen species; SCO2, synthesis of cytochrome c oxidase; TIGAR, TP53-induced glycolysis and apoptosis regulator; Tx, transformed cell.

One of the by-products of an active aerobic metabolic state is the formation of reactive oxygen species (ROS) such as peroxides. These reactive components are produced by lipoxygenases, NADPH oxidases and malfunctioning mitochrondria making errors in complex 1 oxidative phosphorylation. The production of ROS is damaging to a cell causing DNA breaks, oxidizing proteins and lipids and disrupting functions essential for cell maintenance, growth or division. Not surprisingly, ROS activates p53, which in turn transcribes a set of genes that inactivate ROS [29]. For example p53 transcribes a selected set of the sestrin genes, which produce proteins with a number of reduced cysteine residues (R-SH) that destroy ROS by reacting with it resulting in disulfide bonds [30]. Similarly an activated p53 induces the transcription of Parkin and GLS-2 each of which results in higher levels of reduced glutathione (G-SH) [27,28]. ROS reacts with G-SH producing the oxidized form of glutathione (G-S-S-G) and destroying ROS. The p53- regulated gene, TIGAR, produces more glutathione by inhibiting the glycolytic enzyme phosphofructokinase, such that glucose is now shuttled into the pentose phosphate pathway and away from the glycolytic pathway [31]. The pentose phosphate pathway produces NADPH, which is required for generation of reduced glutathione. As discussed above the activated p53 protein shuts down the IGF-1/mTor pathway and the inactivity of TORC1 induces autophagy, which can include the destruction of defective mitochondria (mitophagy). Thus p53 can induce compounds and proteins that inactivate ROS and eliminate defective mitochondria. But p53 has other important effects on cellular ROS (Figure 2). p53 mediated cell death can result from the transcription of BAX, PUMA and NOXA which act at the mitochondria to release cytochrome c. Cytochrome c combines with the p53 regulated gene product APAF-1, which then activates caspases resulting in cell death by apoptosis [6-8]. ROS increases during apoptosis, and so a decreased expression of BAX and PUMA observed with a mutant p53 in cells not only blocks apoptosis but also decreases ROS[32]. Indeed an active p53 can also increase ROS. P53 induces ferrodoxin reductase and PIG3, which are part of ROS-generating pathways [33], and represses Pdk2 resulting in strong ROS production by the electron transport chain [25]. And finally, it should be noted that in response to high ROS p53 can effect mitochondrial integrity directly, by changing its conformation and inserting into mitochondrial membranes, thereby opening a large pore that leads to necrosis [34]. This dual ability of wild type p53, to produce ROS inactivating functions under one condition and to induce ROS levels under different conditions provides a good example of the protective or destructive roles of the p53 protein. It is not clear how these dual roles are regulated nor is it clear what initiates these diverse responses.

## The Parkinson pathway responds to ROS

Over the past few years a number of genes have been identified whose mutant alleles have been shown to contribute to early onset Parkinson's Disease. The functions (at least in part) of four of these genes (PINK-1, PARKIN, DJ-1 and LRRK-2) are to respond to the presence of ROS and eliminate defective mitochondria by mitophagy, reduce ROS levels and even kill cells that are damaged by ROS [35-40] (figure 2). In response to ROS the PTEN induced protein kinase (PINK-1) combines with the ubiquitin ligase Parkin and the complex translocates to the mitochondria [36,37]. Pink phosporylates Parkin which then polyubiquitinates proteins on the surface of the mitochondria initiating mitophagy in response to ROS production [37]. Similarly DJ-1 protein concentrations increase in response to ROS and it is translocated to mitochondria in a Pink-Parkin dependent fashion [39]. Based upon the structure of DJ-1 it is thought to function as a peroxidase inactivating ROS produced in mitochondria. Interestingly the oxidized DJ-1 cysteines are regenerated to reduced (R-SH) residues by glutathione, whose levels are increased by GLS-2 and Parkin, both p53 regulated genes (figure 2). A fourth Parkinsons gene, LRRK-2, is a protein kinase that, unlike Pink, Parkin and DJ-1, (which are recessive alleles resulting in Parkinsons Disease) acts as a dominant mutation responding to ROS by initiating programmed cell death [41]. There is a curious relationship between p53 and DJ-1. In cells with wild type p53, DJ-1 levels are low but respond to the presence of ROS by small increases in the DJ-1 protein concentration (2-3 fold). In transformed cells that have p53 mutations, the DJ-1 levels are commonly very high (30-100 fold increases). This suggests that p53 negatively regulates DJ-1 levels in non-transformed cells. However both transformation and p53 mutation is required to raise DJ-1 to very high levels in cells [42]. This means that DJ-1 is the dominant limitation on ROS levels in p53 mutant cancer cells, while p53 may take a leading role in normal cells with wild type p53. DJ-1 also has a curious relationship with PTEN another major tumor suppressor gene product. In a screen carried out in Drosophila (in the eye of Drosophila) DJ-1 was shown to be a suppressor of excessive PTEN activity [40]. These observations are consistent with the observation that DJ-1 can function as an oncogene and transform cells in culture along with other oncogenes [43]. These observations do not appear to be consistent with the claim that DJ-1 functions as a peroxidase at mitochondria. If DJ-1 is a peroxidase it should protect cells from ROS and reduce cellular damage while lowering the increased mutation rate observed with higher levels of ROS. This is expected for a tumor suppressor gene, not an oncogene.

## What can we conclude and what questions remain?

The integration of some of the gene functions that lead to early onset Parkinsons disease when defective (Pink-1, Parkin, DJ-1 and LRRK-2) into the p53 and PI3K/mTor pathways responding to ROS suggests a possible causation for at least some types of Parkinson's disease. Indeed T. Mak and D. Park and their colleagues have demonstrated using knock out mice that these gene functions reviewed here that are associated with mitochondria and ROS, may well play a role in neurons [38-39]. Why the dopaminergic neurons of the Substantia Nigra should be particularly susceptible to this type of cellular stress is unclear but some have speculated that these neurons have fewer mitochondria than other types of neurons so loss of some to ROS might result in a bigger problem for the cell, lead to ATP limitations and cell death at an earlier time. The relationship between the Parkinson pathway genes and p53 and IGF-1/mTor pathway brings up the question of whether p53 or LRRK-2 could be initiating cell death in these neurons? The possible role of ROS in Parkinson's Disease suggests the use of reducing agents such as N-acetyl- cysteine for treatment by reducing ROS levels [44-45]. There have been clear positive associations between an increased risk of prostate cancer and melanoma in patients with Parkinson's Disease or in individuals who eventually developed Parkinson's Disease [46-47]. At the same time there is a lower risk for smoking related cancers of the lung and larynx in Parkinsons patients even taking into account the smoking habits of the group [48]. Whether this is a reflection of the cancer promoting roles of ROS in different tissues remains to be explored.

The role of Parkin in ROS reduction helps to explain why it is sometimes called a tumor suppressor gene and both alleles can be found in a mutant form in some cancers [49]. It also helps to explain its role in metabolic control of the Warburg effect and its ability to enhance glutathione levels in cells [28]. This is also consistent with the reasons why Parkin is a p53-regulated gene responding to ROS induced stress. It is interesting that mutations in genes that populate these three signal transduction pathways can result in cancers, neurodegenitive diseases and metabolic alterations supporting cell growth and division. This suggests that there are significant differences in the tissue specific uses of these pathways in different cell types resulting in diverse phenotypes depending upon the gene with a mutation in a pathway.

The role of p53 in regulating ROS can be demonstrated by the observation that cancers in p53 knockout mice can be delayed by the administration of N-acetyl-cysteine [44]. This suggests that a mutation in the p53 gene leads to enhanced ROS, which in turn leads to more rapid development of cancers. P53 not only regulates ROS by sestrins, Parkin, GLS-2, TIGAR and by generating enhanced levels of reduced glutathione (R-SH), it can shut off TORC1 and 2 initiating mitophagy [50]. Interestingly two papers have recently appeared demonstrating that treatment of p53 knockout mice or heterozygous mice (as in Li-Fraumeni patients) with rapamycin, a drug that inhibits TORC1, can also slow the appearance of tumors in these mice [51-52]. The absence of (or lower levels of) p53 in p53 mutant mice relieves the break upon the TORC1 pathway and Rapamycin restores that break delaying the progression of mutations required to produce a cancer in either a knock out mouse or Li-Fraumeni (heterozygous) mice. This observation brings up the interesting possibility that treating Li-Fraumeni patients with either N-acetyl-cysteine and/or Torc1 inhibitors might delay or reduce the number of tumors that develop in these patients over a lifetime. These observations are consistent with an important role for ROS in the development of cancers and the progression of cancers. This may especially be the case in cancers that harbor p53 mutations. One of the reasons why cancers with p53 mutations often have a poor prognostic outcome could be because of high levels of ROS in the tumor cells. The implications for the diet and the type of drugs employed to treat cancer patients could be important and these concepts should at least be tested.
